# Analysis of the properties of a glass/sisal/polyester composite

**DOI:** 10.1038/s41598-020-79566-7

**Published:** 2021-01-11

**Authors:** Rivalani B. Baloyi, Sizo Ncube, Mufaro Moyo, Londiwe Nkiwane, Pethile Dzingai

**Affiliations:** National University of Science and Technology, PO Box AC 939, Bulawayo, Zimbabwe

**Keywords:** Materials science, Structural materials, Composites

## Abstract

Composites are gradually becoming an integral material for structural and manufacturing processes. Sisal fibre has the potential to be one of the leading reinforcement natural fibres, not only in Zimbabwe, but the world over for use in glass composites. This is mainly due to it being inexpensive, exhibiting a low density, high specific strength, a high stiffness to weight ratio, non-toxicity, its abundance in Zimbabwe and its renewability. With an aim of coming up with a composite for partition board applications in the construction industry, five samples of sisal-glass composite were prepared with varying sisal fibre content and different layering techniques. Sisal fibres were pre-treated with 20% NaOH to enhance their crosslinking with the resin and reduce the chemical impurities such as lignin and waxes. Mechanical (flexural test, hardness, and tensile strength) and physical tests (density and water absorption) were conducted to analyse the properties of the composite. The results showed that treated sisal fibres had a higher breaking tenacity of 33.11 g/tex which is higher than untreated fibres with a breaking tenacity of 25.72 g/tex. The best properties were obtained in the sample with 4 layers of glass and 9 layers of sisal fibres using 200 ml of resin. The composite had a tensile strength of 57.60 MPa, flexural strength of 36 N/mm^2^, moisture absorption of 10% and a burning rate of 9.82 mm/min. These results were then compared with those of the current partition boards in the market. It was noted that the composite was suitable for use in partition boards. Again, regarding cost, these composites are cheaper to produce at a rate of $11.33/m^2^ compared to the timber costing at $16/m^2^.

## Introduction

The advancements in the field of textiles led to many new and advanced materials. These gave rise to the development of composites materials. Composite materials, in simple terms, are materials that are comprised of at least two different materials with one the materials remaining in a solid state during fabrication^1,2^. Glass reinforced fibre composite materials give the engineer an alternative of performance of materials for the automobile sector and building sector. The increased demand of natural fibre is due the fibres being inexpensive, low density, environmental friendliness, inexhaustible supply and its profusion^3–6^. Due to the lightweight nature of natural fibres and the mechanical properties of natural fibre composites the number of application in the modern industry has increased and even includes transportation and aerospace industries^1,4^.

### Fibres in composites

Sisal fibres are a renewable resource, and the production of this fibre uses a small amount of energy^4–6^. While natural fibres traditionally have reinforced thermoplastic polymers, they have attracted greater attention in reinforcing glass fibres with their added advantage of mechanical properties which are required in automobile applications^7^. Use of sisal fibres to reinforce glass has several advantages such as reduced environmental impact from synthetic fibres and decomposability of components^3,6,8,9^. The most commonly used fibres are called bast fibres or skin fibres. They are located in the phloem of specific plants, mainly dicotyledonous plants. They provide the support to conductive cells and the necessary rigidity required by the stem^6^. Fibres are separated from the plant using a process known as retting. Generally, bast fibres exhibit higher strength when compared to other natural fibres hence their use in high quality textiles. Types of bast fibres found in nature include flax, hemp, jute and kenaf^10^. Bast fibres have been used to manufacture traditional textiles such as carpets, hessian or burlap, paper and sacks.

In more advanced textile materials they have been used to manufacture nonwovens and composite materials that have been used to make products such as mats, furniture materials, door panels for vehicles and carpets^5^. Short conventional fibres such as aramid, glass and carbon have recently found increasing number of products as reinforcement for polymeric materials. The objective of the use of short fibres is to improve the mechanical properties of polymeric material while keeping costs low^11^.

### Natural fibre composites

The capability of the composite is dependent on the matrix as the matrix is responsible for the distribution of the loads on the composite material. Composites are made of a matrix and a reinforcement component. The matrix is important in the performance of the composites. Due to their ease of handling and good bonding between the matrix and fibres, thermosets have been used for the matrix component^12^. Thermosets have the advantage of the ability to be cured to a high degree that results in a 3D network structure that is tough, resists creep and solvents. Due to fibre alignment the loading can be above 70%^13^.

Most of the work has centred on synthetics such as polyethylene, poly vinyl alcohol, polypropylene and polystyrene as they can be easily processed at temperatures below 200 °C, above which natural fibres would be damaged^14^. Dispersion of fibres in thermoplastic composites plays an important part in efficient and even distribution of fibres in composite materials^8^. Although the processability of the composite materials results in ion limited loading, thermoplastics composites exhibit good properties such as desirable toughness and mechanical properties. Fibres in the composite have been found to be of a randomly oriented nature leading to reduced property medication of thermoplastic composites. The resulting properties of the composite material will be determined by the fibre, fibre matrix interface and the aspect ratio of the fibre^8^.

Bonding between the fibre and the matrix is essential in determining the forces that the matrix can withstand. Strong bonding between matrix and fibres means that there will be a high level of force transfer as it moves from the matrix to the fibre, thereby allowing the fibres to effectively perform the load bearing function.

The thermal stability of the sisal fibre is critical in the manufacture of composite materials as high temperatures are experienced during the curing stage of the thermosets^14^. During processing at high temperatures, the walls of the sisal fibre undergo pyrolysis which results in formation of charred layers. These charred layers will then form a protective layer around the lignocellulose to prevent further degradation. Thus the key factors in the fabrication of sisal reinforced composites are thermal stability of the fibre, bonding behaviour of sisal, and distribution of the fibre in the composites^15^.

## Methodology

### Fabrication of the composite

The composite fabrication process was fabricated using the hand lay-up method. Unsaturated orthopthalic polyester resin was used as the polymer for the matrix. The resin with supplied with an accelerator, that is, cobalt napthanate already mixed so the only additive that was added to the resin was the catalyst, Methyl ethyl ketone peroxide (MEKP). Wax was used as the release agent. A designed 400 × 400 mm metal weight (10 kg) was used for the compression of the composite. Plastic brushes and a roller were used for the spreading of resin and removing air pockets respectively.

### Preparation of materials

#### Pre-treatment of sisal fibres

Natural fibres are generally extracted with some percentage of impurities and they contain cellulose and lignin which may prevent them from reacting with other chemicals. In order to remove this and increase the surface reactivity of sisal for cross linkage with the resin, pre-treatment with a NaOH was done in a process known as mercerisation^9^. The Sisal fibres were treated to reduce the water absorbency of sisal fibres and to remove impurities and enhance the surface reactivity of sisal fibres.

The fibres were treated by immersing 200 g of sisal fibres in 20% sodium hydroxide for 1hour at room temperature. The fibres were then rinsed with distilled water then dried in open air for 2 days. Treated fibres were also characterised for their strength properties using an Instron tensometer with a load cell of 3 kN. Obtained values from the test were recorded, averaged and compared to the tensile properties of the untreated fibres.

Treated and untreated sisal fibres were chopped into short lengths of 50 mm. The sisal fibres were randomly laid to produce mats of dimensions 300 mm × 300 mm. Each sisal mat had a mass of 10 g and each glass mat had a mass of 66 g. Each composite specimen consisted of totally 4 layers of glass mats and alternating layers of sisal fibres for the preparation of different samples, as shown in Table 1.

**Table 1:** Sample preparation.

Sample	Arrangement of glass (G) and sisal (S) layers	Number of layers of sisal	Amount of resin used (ml)
1	G|S|G|S|G|S|G	3 (Untreated)	200
2	G|S|G|S|G|S|G	3 (treated with NaOH)	200
3	GG|SSS|GG	3 (Different layering)	200
4	G|SS|G|SS|G|SS|G	6 (Untreated)	200
5	G|SSS|G|SSS|G|SSS|G	9 (Untreated)	200

### Characteristics of the woven glass roving

Glass material which was used was E-glass, woven roving with a density of 450 gsm.The glass fabrics were cut into 5 weft and 5 warp direction specimens according to the dimensions 150 mm × 100 mm as described in the ASTM D5034 standard for grab test. The specimen were then tested for their individual tensile properties using a Titan universal strength tester with a load cell of 3 kN at a rate of 100 mm/s. Load was applied to the test specimen until it broke and values for maximum load and elongation were recorded. The Glass fibres matts were obtained from ACOL Chemicals, Bulawayo.

### Preparation of resin

The matrix used to fabricate the fibre material was preaccelerated orthophthalic polyester with a density of 1.10 g/cc. Curing of the resin was initiated by a catalyst, methyl ethyl ketone peroxide (MEKP) which hardens the fibre composite without external heat. Curing time of the polyester resin was 18minutes from the time it was mixed with the hardener. To prevent hardening before finishing the fabrication process, mixing of the resin was done, using a steel rod, prior to the fabrication. A measured amount of polyester resin was taken for different fibre content and mixed with the hardener in the ratio of 1% w/v. The solution was mixed and stirred before applying on the laminate. The resin used was sourced from ACOL Chemicals, Bulawayo which supplied the preaccelerated orthophthalic polyester from the NCS Resin Company.

## Fabrication of the composite material

A mixture of polyester resin and catalyst was hand laid on polyethylene film covered with a release film to prevent sticking. In this case, a thin layer of wax was used as the release film. The amounts of resin and catalyst used are shown in Table 1. The order of laying the materials is also shown in Table 1. Rollers were used to remove air pockets and allow resin to flow between the fibres. A weight was then placed on the fabricated composite with the polyethylene film placed in-between a 10 kg weight and the composite. Each composite sample had four layers of glass and 200 ml of resin was used for each.

Finally, these samples were left for 24 h for them to cure at room temperature (24 °C) to get the desired samples. After the composite material cured, the composite material was then taken out and post curing was carried out in an oven at 50 °C for 4 h. A laboratory type oven was used for post curing of the composite material.

### Tensile properties

This test method determines the in-plane tensile properties of polymer matrix composite materials. Tests were carried according to ASTM D3039 standard using an Instron Universal Testing Machine (UTM) at room temperature conditions and at a speed of 2 mm/min. 5 specimen were tested, each being averaged from the 5 tests. Specimens used for testing were 250 mm length by width 25 mm.

The formulae that were used to calculate the values of tensile strength, strain, elongation and modulus of elasticity are given below:

1 $$Ftu = Pmax/A$$

2 $$E = \frac{\sigma }{\varepsilon }$$

3 $$Strain =\Delta l/l$$

where *F*tu = ultimate tensile strength, MPa; *P*max = maximum load before failure, N; *A* = Average cross-sectional area (W × H), mm^2^; σ = Ultimate tensile stress; E = Elastic modulus; $\varepsilon$ = strain at maximum load; Δl = change in length produced; l = initial length.

### Flexural properties

The standard used for flexural test was ASTM D790. A DMG Universal Testing Machine was used to carry out the Flexural test. Flexural test determines the maximum stress induced in the outermost fibre.

The flexural strength is calculated from the maximum load of the composite using the following formulas.

4 $$Wb = B.H^{2}/6$$

5 $$Mbmax. = Fmax . L /4$$

where F_max_ = maximum force (N); L = length (100 mm); H = thickness and B = breadth

6 $${\text{Flexural}}\;{\text{strength}}\;\sigma {\text{b}}.{\text{B}} = {\text{M}}_{{{\text{bmax}}}} /{\text{W}}_{{\text{b}}}$$

### Moisture absorption test

ASTM 5229- Is the Standard Test Method that is used for composite materials to determine the Moisture Absorption Properties and Equilibrium Conditioning. Five tests were carried out per sample. The tests for determining moisture absorption were carried out at room temperature.

Percentage water absorption is then calculated from:

7 $$\frac{{{\text{Final}}\;{\text{weight}} - {\text{initial}}\;{\text{weight}} \times 100}}{{{\text{Initial}}\;{\text{weight}}}}$$

### Hardness test

The hardness test was carried out following the procedures of the ASTM F1957 standard. The test characterizes the indentation hardness of materials through the depth of penetration of an indenter. The test is mostly used for composite materials to determine the curing degree of reinforced thermosets. There test also determines the resistance to plastic deformation by a material.

### Flammability test

The flammability test was carried according to ASTM D635 standard. The flammability test carried out to determine the rate at which a material will burn when placed near a flame**.**

## Results and discussion

To obtain the properties of the fabricated composite, five samples of different fibre and matrix mass percentages were prepared. The respective mass percentages were obtained from the weighed masses of the composite using a digital analytical scale. The compositions of the composite samples were calculated using the formulas in the methodology section. From each of the samples, three test specimen were obtained and subjected to bending, tensile, hardness and moisture absorption tests. The results of the fabrication of the composite are shown in Table 2.

**Table 2:** Density and thickness for the composites.

Sample	1	2	3	4	5
Density (g/cm^3^)	1.307	1.311	0.829	0.885	0.712
Thickness (mm)	5	5	6	8	10

### Tensile test results

Tensile test results show how much force the composite can withstand without breaking and from the load-elongation graph the tearing and deformation properties can be obtained. Table 3 gives the summarised maximum load values and extension and other calculated values from the load-extension graph.

**Table 3:** Tensile test results for composite samples.

Sample	1	2	3	4	5
Area (mm^2^)	125	125	125	200	250
Max load (kN)	15.33	16.0	11.87	14.67	14.40
Extension (mm)	6.26	8.86	8.67	8.60	7.90
Tensile strength (MPa)	112.64	128	94.96	73.35	57.60
Strain at maximum load (MPa)	0.025	0.035	0.035	0.034	0.032
Elongation (%)	2.5	3.5	3.44	3.16	3.47

Table 3 shows a comparison of the tensile strengths of the composites sample which were fabricated according to Table 1. The treated fibre composite (2) shows a higher tensile strength of 128 MPa which is higher by 16 MPa compared to that of sample 1, which is comprised of untreated fibres but of similar laying of fibres. Thus, sodium hydroxide treatment is shown to have caused a substantial increase in the tensile strength of the composite. The NaOH removed amorphous parts of the fibres, namely, hemicellulose and lignin which resulted in an increase in tensile strength of the fibres. Removal of the amorphous parts of the fibres also improved interfacial adhesion between the fibres and the matrix as exhibited by a higher tensile strength in the composites made using NaOH treated fibres than those made using the untreated fibres.

Comparing the tensile properties of current partition boards and the fabricated composite board, it can be clearly noted that the composite is stronger in tensile strength compared to soft wood boards which have a tensile strength of 23 MPa^16^. Again, with the medium strength cement board, the least strong composite can withstand a maximum force of 57.6 MPa which is almost nine times more than the fibre cement load of 6.99 MPa and two times that of the HDPE partition boards which have a tensile strength of 27.6 MPa^17^.

**Figure 1:**
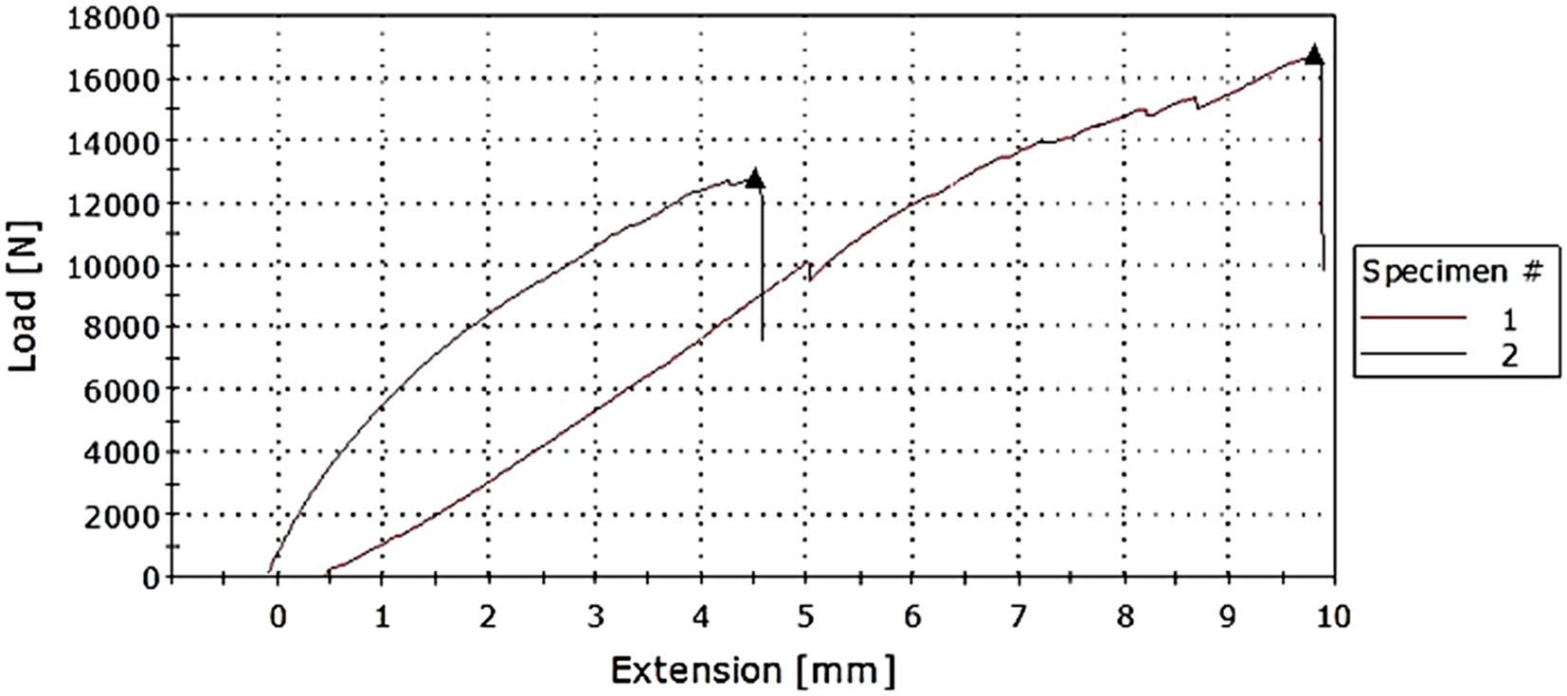
Tensile strength against treated (1) and untreated (2) composites.

Figure 1 shows the relationship between force and extension of the treated and untreated composites. For the major portion of the above force extension curve, the extension of the composite is relatively proportional to the force being applied. That is to say it follows the Hooke’s law. Therefore, the slope of the curve where stress is proportional to strain can be calculated, which is the modulus of elasticity. Increased applied forces result in rapid stretching hence necking or wasting occur beyond the maximum force has been reached after which the final fracture occurs as shown in the tip of the curve.

**Figure 2:**
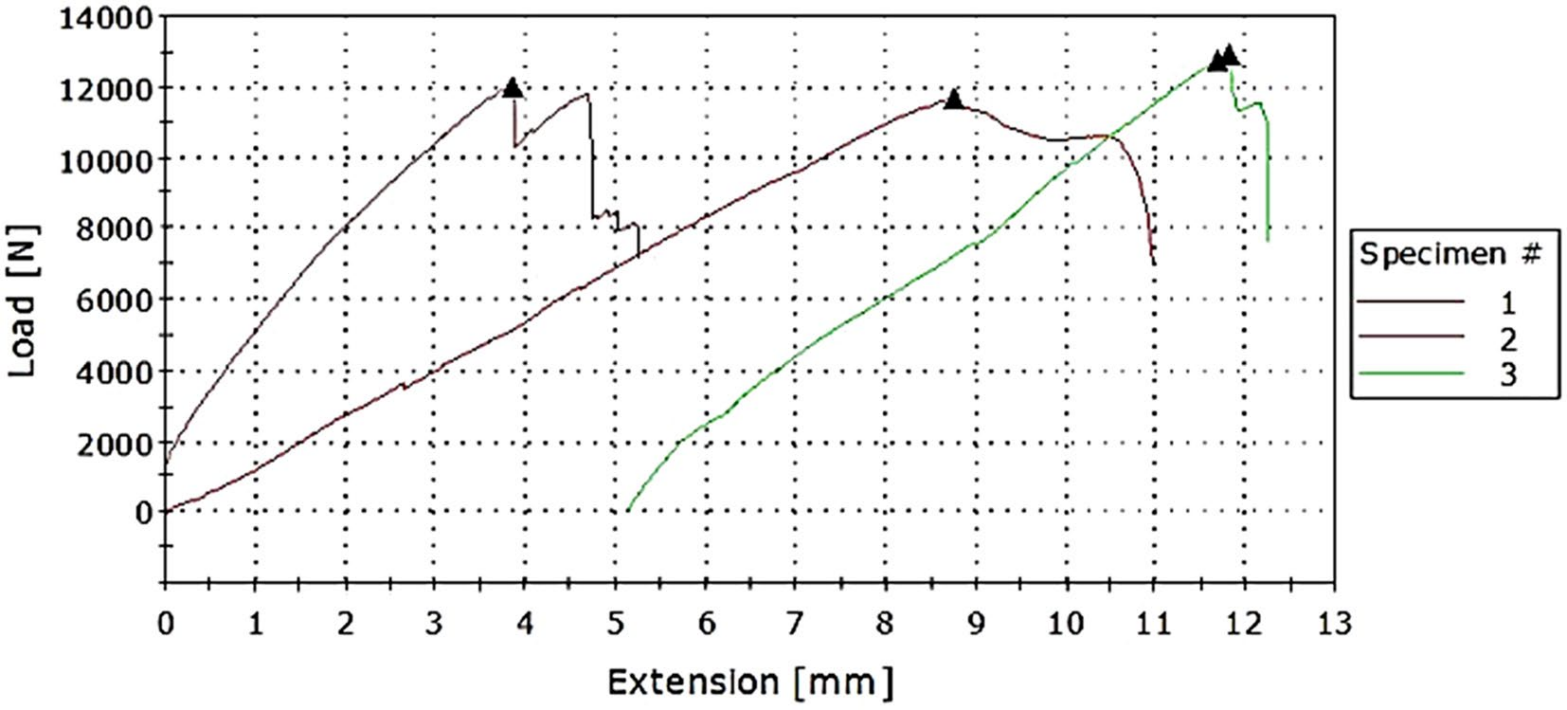
Tensile strength against sisal content (1) G4; S9, (2) G4; S6 (3) G4; S3.

As shown in Fig. 2, increase in the layers of sisal leads to a decrease in tensile strength. The composite with the least number of sisal fibres has more tensile strength. This can be as a result of the crosslinking between the sisal fibres and resin compared with that of glass and resin. If there are less layers of sisal fibres, the bond between the glass layers sandwiching the sisal is stronger than when there are more layers of sisal fibres between. Thus, it will need more load for it to break hence a higher tensile strength. The failure pattern of sample 1 and 3 can be attributed to the bonding between the sisal layers and the resin where the sample laminates initially separated before failing individually. Sample 2 had better bonding as the sisal fibres had been treated.

**Figure 3:**
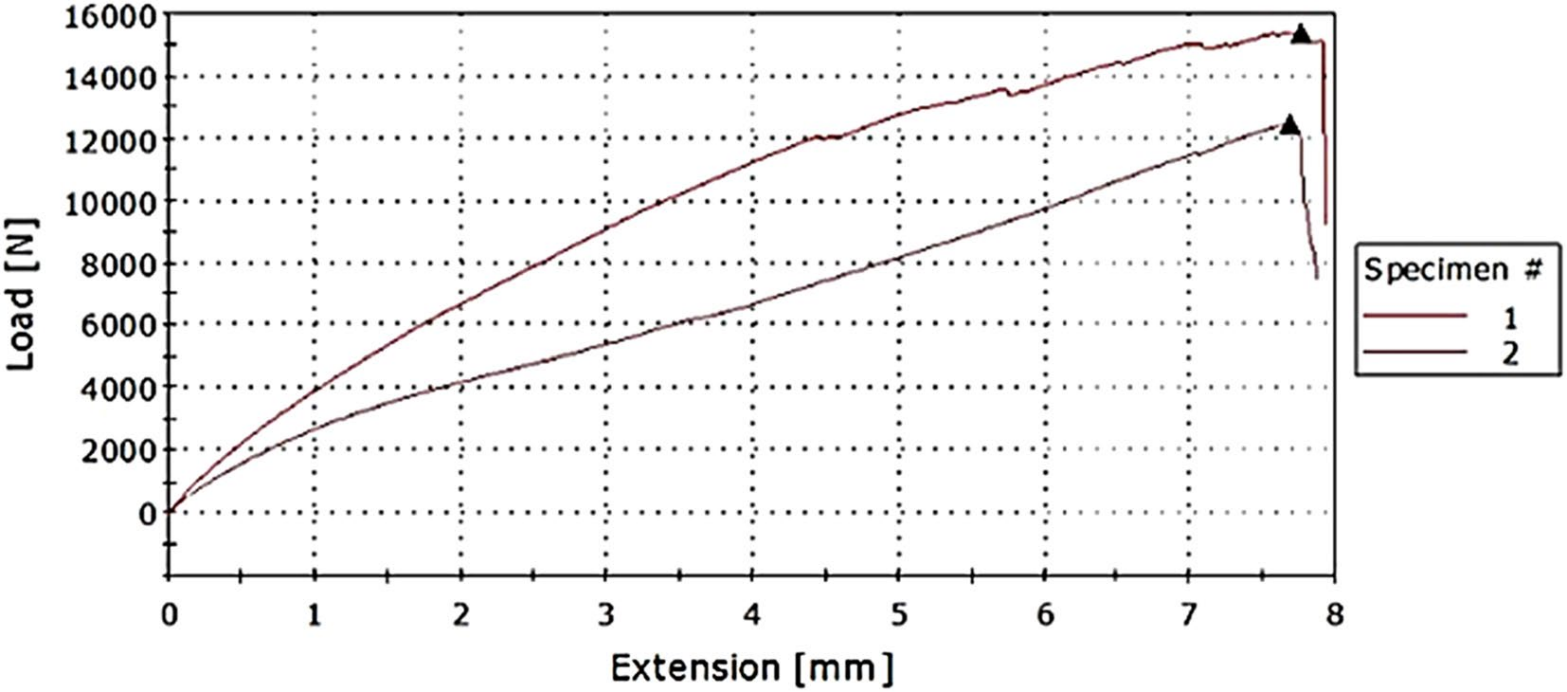
Tensile strength against sample type (1) Alternating (2) All sisal layers in the middle layers.

It is noted from Figs. 2 and 3, that the composite with alternating layers is stronger than the one which has all the sisal fibres sandwiched between outer layers of glass. Strength in sample 1 is more distributed than in sample 2 where the strength in the outer surface is greater than in the middle. This lowers the overall strength of the composite.

### Fracture properties of composites under tension

**Figure 4:**
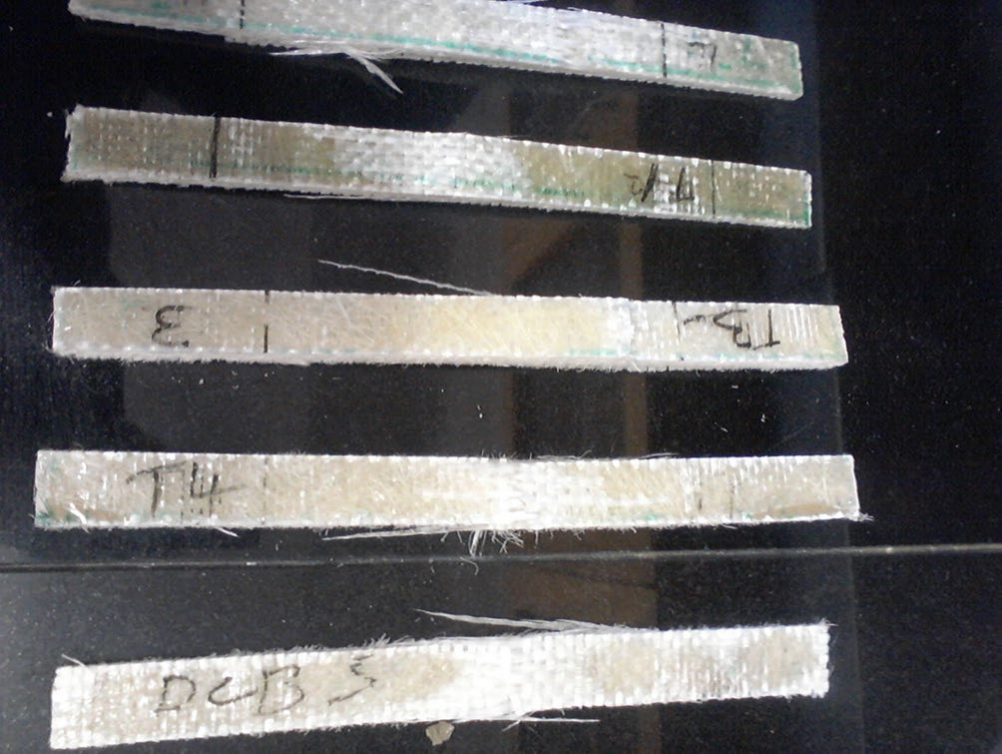
Tearing behaviour of the composite specimen under tension.

As shown in Fig. 4 fracture of short fibre composites will generally occur parallel to the fibre axis of the composite material. For a composite material the strength of the composite will be larger in the direction of the fibres compared to the transverse direction. Fracture of the composite materials may occur in either the fibre-matrix interface or within the matrix instead of the composite surface as shown in the specimen. Fracture behaviour of the composite is also a result of the different layer failing at different stages leading to a high and low yield strength.

### Bending test results and calculations

Using sample 1, the tabulated results in Table 4 were obtained using the maximum load obtained from the bending test using Eqs. (4), (5) and (6). The rest of the results were calculated and tabulated as shown in Table 4 using the averaged maximum load values, samples length and width was 100 mm and 20 mm respectively.

**Table 4:** Bending test results.

Sample	F_Max_ (N)	H (mm)	W_b_ (mm^3^)	M_bmax_ (Nmm)	δ_b_ (N/mm^2^)
1	520	5	83.333	13,000	156
2	615	5	83.333	15,375	184
3	170	5	83.333	4250	51.00
4	420	8	213.333	10,500	49.22
5	490	10	333.333	12,250	36.75

Flexural stress of the composite with the highest flexural strength is 156 N/mm^2^ and its deflection at break is 5.0 mm. this is because it is more brittle compared to the other specimens hence it quickly reaches the maximum load without too much deflection.

**Figure 5:**
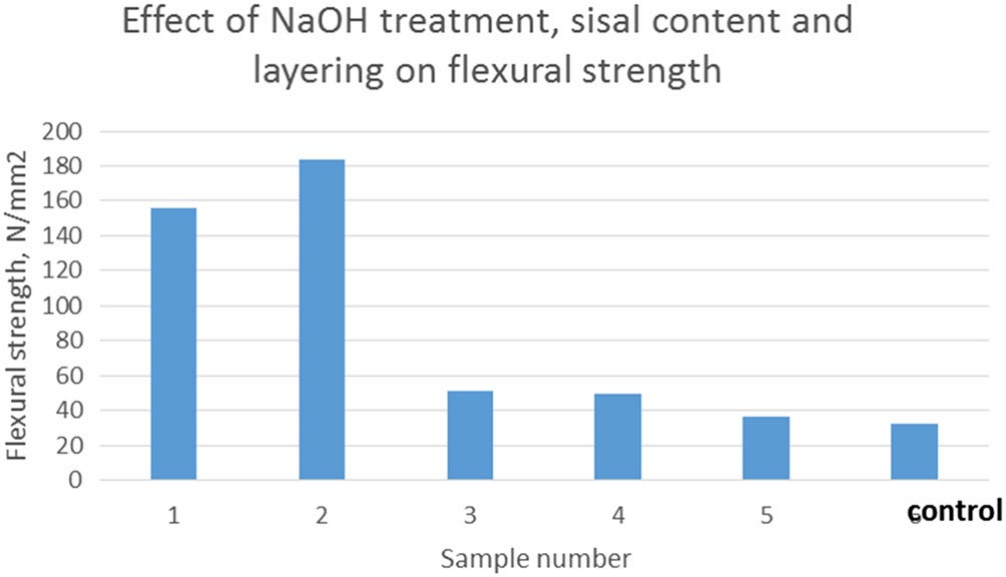
Flexural strength against sample type.

As shown in Fig. 5, treated composite (2), composed of three layers of sisal treated with NaOH, has flexural strength of 184 N/mm^2^ which is higher than that of untreated composites.

The interfacial zone between two surfaces in a composite material, namely between the fibres and the matrix plays an important part in the performance of the composite. Load transfer will depend on the interfacial zone between the fibre and the matrix thereby affecting strength characteristics of the composite. On the other hand, flexural failure will be determined by the bonding between the fibre and the matrix. Increase in the flexural strength can be attributed to the increase in effective surface area available to the matrix to bond. Flexural strength decreases with increase in sisal fibre content shown by the decrease in the graph size from sample 1 to 5. Due to the random orientation of the sisal fibres, more layers will cause more weak areas which are not uniform in their bonding strength, hence there is presence of more weak spots in the composite. Deformations can occur at lower loads than a composite with low sisal fibre content.

A composite with alternating layers of glass and sisal, that is comparing sample 1 and 3, has a higher flexural strength than that which has sisal fibres concentrated in the middle portions of the composite (sample 3). The difference in strength of the two fibres used will cause the composite to be stronger in the outer layers than the core area. Hence when a bending force is applied the core is likely to start deforming and cause the outer layers to break also. The sample with the lowest flexural strength, that is, sample 5 with a maximum bending load of 490 N has greater flexural properties than that of the current gypsum board which has the same thickness, that is, 489N^18^. Compared to that of wood partitions the composite has also higher flexural properties than wood, which is 32 N/mm^2^^17^.

### Breaking tenacity

Tests carried out for breaking tenacity showed that there is a higher breaking tenacity for the treated fibres as compared with the untreated fibres. Samples with treated fibres showed an average breaking tenacity of 33.11 g/tex while samples with untreated fibres had an average of 25.72 g/tex. Treatment with NaOH (mercerisation), allows for even distribution strength properties along the fibre length as it increases the lowest strength that the fibre can withstand at any point along its length^19^. Mercerisation also improves absorption of chemicals by the fibre by opening the lumen of the sisal fibres thereby allowing exposure of reactive sites. The opening of the reactive sites allows crosslinking of the resin and the fibre leading to better bonding, hence an increase in the strength of the composite material.

### Moisture absorption test

Table 5 shows the average weights of the initial and final samples.

**Table 5:** Moisture absorption test results.

Time	1 h			5 h			24 h		
Sample	Original mass(g)	Final mass (g)	Water Absorbed (%)	Original mass(g)	Final mass (g)	Water Absorbed (%)	Original mass(g)	Final mass(g)	Water absorbed (%)
1	13.94	14.58	**4.46**	14.01	14.70	**4.96**	15.02	15.68	**4.38**
2	14.96	15.58	**4.12**	15.07	15.73	**4.39**	14.07	14.59	**3.69**
3	14.31	15.94	**7.61**	14.42	15.51	**7.59**	14.44	15.42	**6.79**
4	15.51	17.21	**10.96**	17.60	19.27	**9.40**	17.70	18.96	**7.11**
5	19.71	21.70	**10.11**	19.69	21.67	**9.81**	19.74	21.78	**10.34**

**Figure 6:**
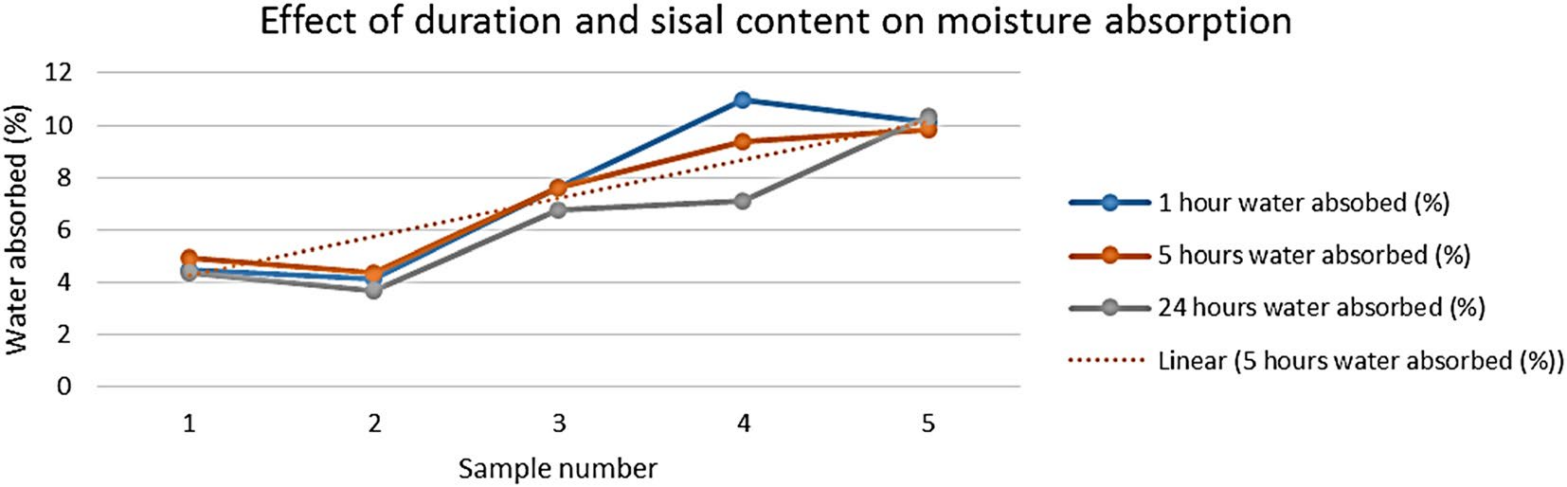
Effect of duration on moisture absorption.

The trend line in Fig. 6 shows that water absorption increases with an increase of sisal fibre content, this is because sisal fibres are fibrous and have more amorphous regions, hence they can absorb water and since there is increase in sisal fibre content in composite there is higher water absorption rate. Hardened polyester resin absorbs little amount of water.

The composite has an average water absorption percentage which is far less than that of sisal fibres and that of glass, separately. This is due to the use of the polyester resin which also absorbs little water. The composite absorbs less moisture compared to wood partition boards which has a moisture absorption of 12% which is also 2% less than the highest absorption value of the composite^16^. Gypsum boards of 6 mm, which is close to sample 1, have a moisture percentage of 10% yet the composite is only 4.5%^18^. This shows that the composite has good properties than the currently used boards in terms of their water absorption^20^.

### Hardness test results

These tests determine the resistance of the composite to deformation caused by low impact forces such as indentations.

**Figure 7:**
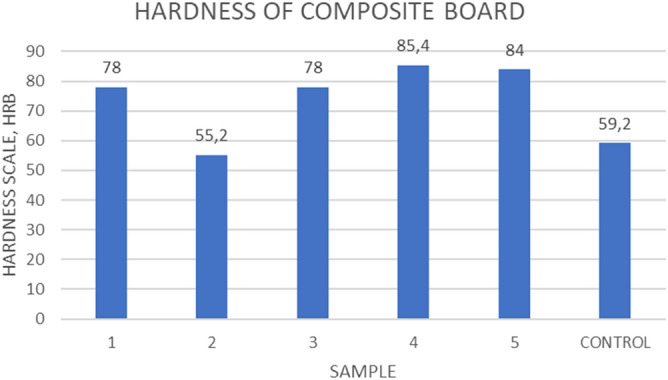
Effect of fibre content and treatment on Rockwell hardness.

From Fig. 7, it can be seen that increase in volume ratio of sisal fibres improves the hardness of the composite. Treated fibre composite reduces the hardness as depicted by the bar for sample number 2 at 57.2 HRB whereas sample 4 has the highest value of 85HRB. Hardness of the sample with 3 layers of sisal but different layering as compared to (1) has the same hardness (78 HRB) as that of sample (1) despite the difference in arrangement.

The composite material is considered a hard material according to the classes of the ASTM standard F1957. Wood partitions are generally made from softwood, such as Pine wood^16^. It is a soft material when comparing its hardness properties to composite. It is easily scratched and leaves marking of low force indentions.

### Burning test

The behaviour of burning of the composite at various fibre layers was observed according to ASTM D 635-76 standard. The results of duration of burning are given in Table 6.

**Table 6:** Results of the burning test.

Sample	1	2	3	4	5
Duration (min)	5.02	4.54	4.36	9.12	10.18
Speed mm/min	19.92	22.02	22.94	10.96	9.82

**Figure 8:**
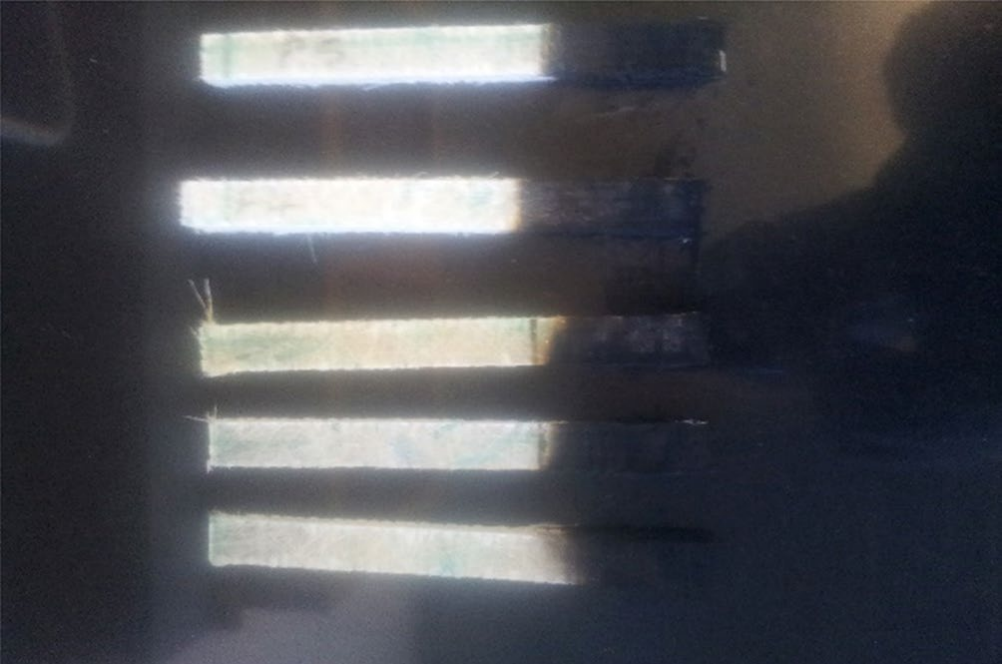
Remains of the samples after the burning test.

The following observations were made during the burning:

- The composite burnt with an orange flame producing a black soot with a chocking polyester/plastic smell
- The composite did not easily catch fire as it took 6 s but burnt easily after catching fire.
- The remains were black in colour with hard black particles as shown in Fig. 8.

Wood partitions are prone to fire hazards. They quickly catch fire and burn readily leaving white ashes. With the composite, it is slow at catching fire and the burning characteristics shows that it takes longer for the fire to spread.

## Conclusion

The current and future application of sisal fibre in composite materials, specifically polymer based composites were explored. The low density and high specific properties of the composite material fabricated in the article, the composite material may find use in vehicle interiors. A sisal-glass composite was successfully developed. With the sample with 4 layers of glass mats and 9layers of sisal being the one preferred since it has the overall thickness which is almost that of the standard partitioning board, that is, 10 mm. Comparing the mechanical properties of the current timber boards in use, the composite has tensile strength and flexural strength which is greater by 32.2 MPa and 4.63 N/mm^2^ respectively. This translates to 239% Also comparing the water absorption properties, the composite has a value of 10% on average which is 2% less that of wood. Mercerisation of sisal fibres results in changes in the structure of sisal fibres which lead to improvements as results show that the composite with treated fibres have higher mechanical properties though there is no difference in the burning properties. The removal of surface impurities on plant fibres may be an advantage for fibre to matrix adhesion as it may facilitate both mechanical interlocking and the bonding reaction due to the exposure of the hydroxyl groups to chemicals such as resins and dyes. However, sisal untreated fibres also show higher mechanical properties compared to wood, hence they can be used untreated to produce the composite without additional costs.
